# Epidemiology of Bovine Hemorrhagic Bowel Syndrome in Belgium and The Netherlands

**DOI:** 10.3390/ani14010107

**Published:** 2023-12-27

**Authors:** Bert De Jonge, Bart Pardon, Jeroen Dewulf, Evy Goossens, Jasper het Lam, Evert van Garderen, Stefan Roels, Jozefien Callens, Thierry Petitjean, Koen Chiers

**Affiliations:** 1 Department of Pathobiology, Pharmacology and Zoological Medicine, Ghent University, 9820 Merelbeke, Belgiumkoen.chiers@ugent.be (K.C.); 2 Department of Internal Medicine, Reproduction and Population Medicine, Ghent University, 9820 Merelbeke, Belgiumjeroen.dewulf@ugent.be (J.D.); 3 Royal GD (Animal Health Service), 7418 EZ Deventer, The Netherlands; 4 Animal Health Service-Flanders (DGZ), 8820 Torhout, Belgium; stefan.roels@sciensano.be (S.R.);; 5 Pathology Department, Arsia Asbl, 5590 Ciney, Belgium

**Keywords:** jejunal hemorrhage syndrome, dairy, hematoma, epidemiology

## Abstract

**Simple Summary:**

Hemorrhagic bowel syndrome (HBS) is a poorly understood, sporadic and often fatal disease in cattle. Although HBS is considered an important disease in dairy cattle, studies about the occurrence and the distribution of this disease in the population (epidemiology) are lacking. This study describes the epidemiology of HBS in Belgium and the Netherlands, based on necropsy records from 2009 to 2022, and reports characteristics about cows and dairy farms with HBS through a questionnaire. A total of 291 confirmed HBS cases in 259 herds in Belgium and the Netherlands were enrolled. There was a significant increase in the annual incidence of HBS in both individual cows and herds from 2009 to 2022. In most recent years, about 1 in 30 necropsied dairy cows died because of HBS. The incidence was higher in the Netherlands compared to Belgium, possibly due to larger herd size. In fall, HBS occurrence was most common. In 35 Flemish dairy herds, HBS mainly manifested as individual cases, with a very variable time between consecutive cases. Most affected cows were in more than 100 days of lactation. In conclusion, HBS is a relevant disease in dairy cattle in Belgium and the Netherlands, with potentially rising occurrence.

**Abstract:**

Hemorrhagic bowel syndrome (HBS) is a poorly understood, sporadic and often fatal disease in cattle. Although, HBS is considered an important disease in dairy cattle, epidemiological data is largely lacking. This study describes the epidemiology of HBS in Belgium and the Netherlands, based on necropsy records from 2009 to 2022, and reports characteristics from 27 cows and 35 dairy operations with HBS, gathered through a survey. The annual incidence of HBS has a significantly increasing trend both at cow and herd level, with incidence above 3.2% in necropsied mature dairy cattle in the most recent years. Estimated herd-level incidence in the Netherlands was double the estimated incidence in Belgium, which might be explained by higher herd size in the Netherlands. Occurrence of HBS was most prevalent in fall, being 40.1% higher than the average of the other seasons. In 35 Flemish (Belgian) surveyed dairy herds with HBS, manifestation of HBS was mostly as solitary cases, and if multiple cases occurred, the time interval was highly variable. In addition, the majority of cows with HBS (61.1%; 16/26) were in more than 100 days lactation. In conclusion, HBS is an important and possibly emerging disease in dairy cattle in Belgium and the Netherlands.

## 1. Introduction

Hemorrhagic bowel syndrome (HBS), often referred to as jejunal hemorrhage syndrome (JHS), is an acute, small intestinal disease of mature cattle with unknown etiology [1]. It is a low morbidity—high mortality disease, well-known among Belgian and Dutch dairy practitioners. HBS typically affects dairy cattle, with Brown Swiss being overrepresented in several studies [2,3]. Nevertheless, occurrence in beef cattle is reported [4]. For almost two decades, HBS was seen as some form of enteritis, with *Clostridium perfringens* type A receiving the most attention as a possible etiologic agent [5,6,7]. More recent work shows that HBS is characterized by an intramucosal hematoma dissecting the muscularis mucosae, which most likely develops from mucosal erosions [8]. This hematoma obstructs the intestine and ruptures, causing severe blood loss with subsequent characteristic blood clot formation in the intestinal lumen. Affected animals show signs of intestinal obstruction with severe weakness, in most cases followed by rapid clinical deterioration, resulting in death within 12–48 h if left untreated [9]. Clinical diagnosis is often challenging, but HBS is easily recognized upon laparotomy or necropsy [3,9], making these the only way of confirming the diagnosis. Even with surgical treatment, the prognosis is guarded and re-occurrence after recovery is common [2,5,10].

HBS is reported in all regions of the United States [5,11,12,13], Canada [10], Europe [3,7,14], the Middle East [15] and Japan [16]. Occurrence is typically sporadic, but outbreaks have been described [5,14]. According to a large-scale study by the United States Department of Agriculture (USDA), herd-level incidence of HBS in U.S. dairy operations in 2002 was estimated at 9.1% and 5.1% having at least one case in the previous five years or preceding 12 months, respectively [17]. Herd incidence for having at least one case was 6.4% in dairy herds with less than 100 cows, while for herds with 100–499 animals or >500 animals, incidence is estimated at 13.4% and 31.2%. In addition, a higher incidence in winter months (38.1%) was reported [17]. Furthermore, management practices implemented to achieve high milk production, have been associated with HBS [12]. Multiparous cows in the first 100 days of lactation are most frequently affected [11,12]. Despite HBS being a globally occurring and important disease for the modern dairy industry, epidemiological reports are sparse, consisting of only two older papers from the United States [11,12]. In Berghaus et al. (2005) and the accompanying study by the USDA [12], the case definition may be not adequate because case farms were identified based on the familiarity of the producer with HBS, especially because only 3.5% had some familiarity with HBS. The other study was based on a small-sized survey of Minnesota bovine practitioners with confirmation of HBS upon laparotomy or necropsy [11]. To the authors knowledge, no epidemiological studies are available from outside the United States nor any report more recent than 2005.

The objective of the present observational study was to describe the temporal and geographical epidemiology of HBS in Belgium and The Netherlands over a 14-year time frame using diagnosis by necropsy as case definition. In addition, in a subset of herds, a survey was performed to describe HBS in more detail.

## 2. Materials and Methods

### 2.1. Study Design and Data Sets

The case definition in this study is diagnosis by post-mortem examination because visualization of the hematoma is essential to confirm HBS. In order to assess incidence on herd and cow level in Belgium and the Netherlands, data from databases of all Belgian (Animal Health Service-Flanders (DGZ), Association Régionale de Santé et d’Identifiaction Animales (Arsia)), and all Dutch (Royal GD) non-university veterinary necropsy facilities, as well as the Laboratory of Veterinary Pathology of Ghent University, were obtained. Royal GD has nationwide coverage in the Netherlands, and Arsia and DGZ receive cases from Wallonia (south part of Belgium) and Flanders (north part of Belgium), respectively. With exception to Arsia, in all diagnostic laboratories, necropsy is supervised by board certified pathologists (ECVP). Data collected comprised date, herd location (postal code), breed type and age for each case of HBS from 2009–2022. If cases were from the same postal code, it was indicated if cases were from the same herd or not, without disclosing the address. Also, for every year and every month, the number of necropsied dairy cattle >2 years old were registered (animals at risk for HBS). Necropsy records were available since 2009 from Royal GD (Netherlands), and from 2009, 2011, 2016 onwards for the different Belgian diagnostic facilities (respectively Ghent University, DGZ and Arsia). No formal ethical approval was needed according to the ethical committee of the Faculty of Veterinary Medicine and Bioengineering from Ghent University, since no protected information was collected and all animals were examined post-mortem.

### 2.2. Cross-Sectional Survey

In order to describe characteristics of HBS in herds and cows in more detail, a second data set was created with herd related information obtained from Flemish herds (n = 35) through a telephone survey. Flanders is the Northern, Dutch-speaking part of Belgium. These 35 herds consisted of 27 herds with a diagnosed case at a Flemish diagnostic laboratory. Diagnosis in the other 8 herds was made by necropsy from the own private veterinarian at the farm (7/8); one animal underwent laparotomy at the university clinic of Ghent university (1/8) and went back to the dairy. Each of the dairy producers were contacted by telephone and informed about HBS and the current study. If the producer agreed, further questions were asked. The following information was collected about the herd at the time of the HBS occurrence: rolling herd average milk production (RHA), herd size, number of diagnosed cases, number of suspected HBS cases and time interval between multiple cases. A lactating cow displaying sudden milk drop, bloody feces, lethargy, anorexia and rapid deterioration was considered a suspected HBS case. In addition, to describe individual animal characteristics, data were collected about the cow with HBS during the same phone call if HBS occurrence was recent as to avoid recall bias (within 72 h, n = 27). Data collected about the cow with HBS included: age, lactation number, days in milk, most recent milk production before disease, interval between first clinical signs and death, observation of dominant eating behavior, observation of bloody feces and manner of death (euthanasia/spontaneous). If animals were euthanized, it was because of severe clinical condition and unfavorable prognosis. The producer was also asked if a new corn/grass silage was recently (≤3 weeks) opened, or another recent change in feeding composition was implemented. Also, the maximal number of plastic covered grass and corn silages at the farm each year were registered. Replacement, addition or any change in the amount of feed components were regarded as a change in feed composition.

### 2.3. Data Analysis

The incidence at cow level was calculated by dividing the number of cases by the total number of necropsied dairy cattle >2 years old (animals at risk) and total dairy population in a certain country, respectively, within a defined period. The incidence at herd level was calculated as the number of diagnosed herds with HBS relative to the total number of herds within a country, in a time interval. In order to identify the association between HBS incidence and year or season, a multivariate logistic regression model was used (MASS package in R v4.2.1). No seasonal information was available on herd-level for the denominator. Therefore, the link between HBS incidence on the herd level and year was identified using univariable logistic regression. For testing difference in incidence over time or between countries, a chi-squared test was used. For all tests, a *p*-value ≤ 0.05 was considered significant. When incidence at cow and herd level is compared between Belgium and the Netherlands, only data since 2015 is used, since there is no data availability for Arsia (diagnostic laboratory of Southern Belgium) before that year. When yearly incidence is reported, years where no data was available from a diagnostic laboratory, the cow population and herds derived from regions which submit cases to this laboratory were not taken into account for incidence calculations. For yearly incidence calculations and geographical epidemiology, only cases derived from the non-university diagnostic laboratories (Royal GD, DGZ, ARSIA) were included to avoid bias as the non-university diagnostic laboratories have a national coverage, whereas the university laboratories have a more regional coverage, which could lead to an overrepresentation of cases from the involved regions. To compare incidence between Belgium and the Netherlands, a correction for necropsy case submission to dairy population was done. Incidence in Wallonia (southern part of Belgium) was multiplied by 1.9 as the necropsy caseload in the Netherlands and Flanders is 90% higher, relative to the dairy cow population. All herds with registered dairy cows were taken into account. The RHA and the herd size at Flemish farms with HBS were compared with the Flemish average at the same year of HBS occurrence, with a two-sided unpaired *t*-test. Flemish averages were retrieved from CRV cooperative and Landbouw Vlaanderen, respectively. The annual average herd sizes of Belgium and the Netherlands were used to estimate the incidence on herd level. These data were retrieved from Statbel (Belgium), Landbouw Vlaanderen (Belgium) and Statistics Netherlands (CBS). For seasonal prevalence calculations, January, February and March were regarded as winter, April, May and June as spring, and so on. Statistical analysis was performed in R (v4.2.1) and GraphPad Prism 8^®^. Data collection and incidence calculation were performed in Microsoft Excel 2019^®^; figures were made in GraphPad Prism 8^®^.

## 3. Results

### 3.1. Description of the Data Set

Between 2009 and 2022, data from 291 cases of HBS were retrieved, derived from 270 herds, with 17 herds having two diagnosed HBS cases and two herds having three. In 283 cases, diagnosis of HBS was established in a necropsy facility. In seven animals, necropsy was conducted at the dairy by a local veterinarian; one animal went back to the farm after laparotomy at the university clinic of Ghent University. A total of 11,263 dairy cattle >2 years old were necropsied from 2009 to 2022 in the veterinary diagnostic laboratories in the Netherlands and Belgium. The yearly caseload at each facility was on average 632.5 ± 152.9 (mean ± SD), 159.0 ± 30.8, 45.9 ± 24.7 and 10.3 ± 5.8 for Royal GD, DGZ, Arsia and the veterinary pathology laboratory of Ghent University, respectively.

The majority of cases from diagnostic laboratories were from the Netherlands (77.5%) with the remaining (22.5%) from Belgium. In 97.9% (277/283) of cases with known breed type, animals were dairy cattle. The other 6 were beef cattle, with 3/6 being bulls. These were the only male animals in the data set, resulting in 99.0% females (287/290). Breed information was available for only 42 Belgian cases, 95.2% (40/42) were Holstein Friesians, and the other 2 cases were Belgian Blue. The mean age of affected animals with known age (n = 266) was 4.2 ± 1.8 years, ranging from 2–9 years.

### 3.2. Temporal Epidemiology

**Figure 1:**
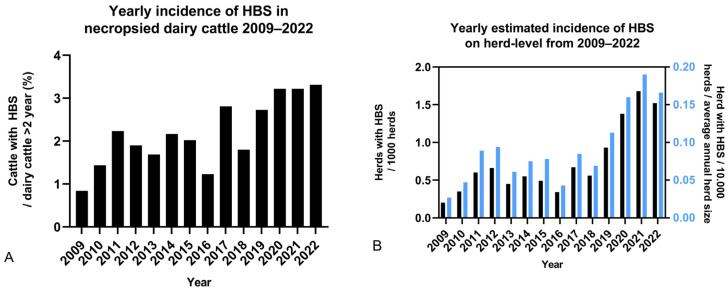
Annual incidence of hemorrhagic bowel syndrome (HBS) in Belgian and Dutch necropsy facilities combined from 2009–2022. (**A**) incidence of cattle with HBS relative to necropsied dairy cattle >2 years old (n = 261). (**B**) Incidence of herds with HBS (n = 259), relative to the total number of herds (black), and the annual average herd size (blue) in Belgium and the Netherlands. In both graphs, an increasing trend can be observed.

The incidence of HBS in necropsied dairy cattle >2 years of age, at the different non-university diagnostic facilities from 2009 to 2022, was 2.2% (261/11,130). The average yearly incidence of HBS in necropsied cattle from 2009 to 2022 was 2.2 ± 0.7%, ranging from 0.8% to 3.3%. There is an increasing trend over the years, with incidence in the second half 50% higher, compared to the first half of the analyzed time period: 1.8% (82/4512) from 2009–2015 compared to 2.7% (179/6618) from 2016–2022 (*p* = 0.0024). From 2010 onwards, incidence was higher than 1% and surged in the last three years to over 3% (Figure 1A). The odds of HBS occurrence in necropsied mature dairy cattle significantly increased every year (incidence in necropsied dairy cattle: OR: 1.09, 95% CI: 1.06–1.13, *p* < 0.0001).

At herd level, the estimated average annual incidence from 2009–2022 was 0.7 ± 0.4 affected herds per 1000 herds in Belgium and the Netherlands. There is an upward trend, with incidence in the second half of the examined period (incidence 2016–2022: 1.01 herds/1000 herds) more than twice the incidence in the first half (incidence 2009–2015: 0.49 herds/1000 herds) (*p* < 0.0001). When the average herd size for each year is taken into account, a similar progression can be observed (Figure 1B). Also on the herd level, the odds of diagnosing an HBS herd in the total herd population increased each year (OR: 1.14, 95% CI: 1.10–1.18, *p* < 0.0001).

**Figure 2:**
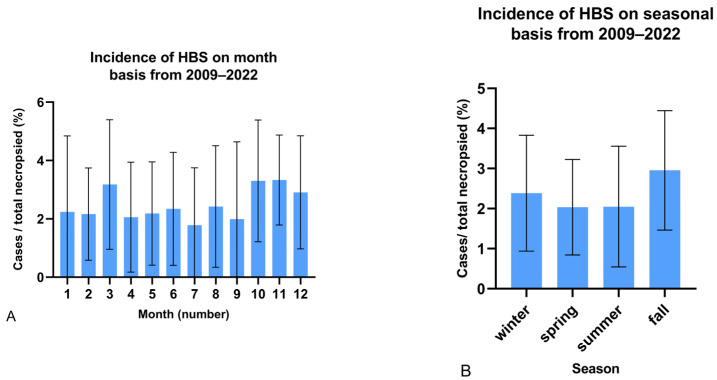
Monthly (**A**) and seasonal (**B**) incidence of hemorrhagic bowel syndrome (HBS) in necropsied dairy cattle >2 years old from 2009–2022 (n = 291). January to March is regarded as winter, April to June as spring, etc. Error bars = standard deviation.

On a monthly basis, the average incidence of HBS in necropsied dairy cattle >2 years, from 2009–2022 was 2.3 ± 0.5%, ranging from 1.7 ± 1.9% in July to 3.1 ± 1.7% in November. Months with the lowest incidence (<2.1%) were February, April, May and July, while the months with the highest incidence (>2.9%) were March, October and November (Figure 2A). On a seasonal basis, fall has the highest incidence of HBS (3.0 ± 1.5%), followed by winter (2.4 ± 1.4%). During fall, the odds of HBS were significantly higher compared to during spring (OR: 1.42, 95% CI: 1.03–1.97, *p* = 0.036) or summer (OR: 1.42, 95% CI: 1.03–1.98, *p* = 0.033). Incidence in spring and summer was about one-third less compared to the fall months, and was 0.2 ± 1.1 and 0.2 ± 1.5, respectively (Figure 2B). No significant difference in HBS occurrence during winter compared to any other season was observed.

### 3.3. Geographical Epidemiology

The overall estimated yearly incidence of HBS in dairy cattle from 2015–2022 in Belgium and the Netherlands, based on necropsy records, was 1.2 HBS cases per 100,000 cow-years. When adjusted to the caseload difference between Belgium and the Netherlands, estimated incidence was identical. When compared to Belgium, estimated incidence in the Netherlands was 25.7% higher (1.3 compared to 1.0 cases per 100,000 cow-years) (*p* = 0.213). However, when corrected for the difference in necropsy case submission (90% higher in the Netherlands and Flanders, compared to Wallonia, the southern part of Belgium), estimated incidence in Belgium was 1.1 cases per 100,000 cow-years. 

At the herd level, the estimated incidence was 1.0 herd per 1000 herd-years from 2015 to 2022 in Belgium and the Netherlands, as well as when difference in caseload was taken into account. Even when the caseload was taken into account, herd incidence was almost twice as high in the Netherlands compared to Belgium (*p* < 0.0001), at 1.2 and 0.6 herds per 1000 herd-years, respectively. In addition, the average herd size in the Netherlands was 66.4% higher compared to Belgium: 103.0 compared to 62.6 mature dairy cows per herd.

### 3.4. Herd and Cow Characteristics of HBS in Flanders (Survey)

Data from a subset of 35 Flemish herds with HBS could be retrieved. All cases occurred between 2013–2022. These dairies had a median RHA of 10,199 ± 1191.5 kg milk per cow, being on average 958.4 ± 208.0 kg higher than the Flemish average at the year of occurrence. The herd size was on average 129.9 ± 79.0 adult cows, which is on average 32.7 ± 13.1 higher than the mean at the year of occurrence in Flemish dairy specialized herds (Table 1). The surveyed HBS herds were significantly higher in RHA and herd size compared to the average Flemish dairy operation (*p* < 0.001). In 74.3% of dairy farms (26/35), it was the first confirmed HBS occurrence ever, with the remaining 25.7% (9/35) having multiple cases. If multiple HBS cases occurred, the median number of confirmed cases was 2.7 ± 0.9 with an average time interval of 137.6 ± 114.3 days (Table 1). In 25.7% (9/35) of herds, other suspected HBS cases were seen at the farm. No diagnosis was established because animals were not necropsied after death, or were sent for slaughter. In 33 HBS cases originating from 29 dairies, information was available about nutritional changes at the time of HBS occurrence. In 69.7% of herds (23/33), a recent feed change occurred in the 3 weeks before the HBS occurrence. A new corn silage or grass silage bunker was opened in 30.3% and 33.3%, respectively, or another feed change occurred (39.4%) (Table 1). Presence of feed change was not registered for two operations.

Additional information from 27 cows with HBS could be obtained and is summarized in Table 2. All originated from different Flemish herds with occurrence between 2020–2022. The mean age was 3.9 ± 1.4 year, with 85.2% (23/27) being higher than the first lactation animals. Lactating cows were in peak, mid or late lactation in 38.5% (10/26), 23.1% (6/26) and 38.5% (10/26), respectively. The interval between first observation of disease and death was shorter than 48 h in 73.1% of cases (19/25), with spontaneous death occurring in 52% of cases (13/25). Bloody manure was noticed in 76.9% of cows (20/26). The observation of dominant eating behavior by the producer was only seen in 15.4% (4/26) of animals.

## 4. Discussion

The case definition in this study was diagnosis by necropsy. This is because HBS is impossible to diagnose with certainty without visualization of the typical gross lesion in the intestines. This is different to the previous work by Berghaus et al. (2005), where familiarity of the dairy producer with a description of the disease was used as case definition [12]. The current study is the first to describe epidemiology of HBS outside the United States.

This study was subject to a number of limitations. The reported (estimated) incidences are underestimations. This is a consequence of our case definition, as obviously no surviving animals are included and only a minor portion of all dead cows are submitted for necropsy (submission bias). Also, it is likely that when HBS is a recurring problem at a dairy, no additional cases are sent for necropsy because the disease is already clinically recognized by the dairy producer and local veterinarian (submission bias). Therefore, herd incidence is a probably a more reliable indicator in this study. In addition, necropsy submission behavior could be different between farms based on herd size and other factors. Furthermore, for the non-university laboratories, necropsy cost and service are independent of the distance to the diagnostic laboratories, so no bias should be expected caused by herd–laboratory distance. At last, data in this study is based on medium-sized, family-owned dairy farms, which are typical for Belgium and the Netherlands, and also for most of Europe. Despite the above-mentioned limitations, bias impacts all data in this study, making it possible to compare incidence of HBS over time and between countries.

The first aim of this observational study was to describe the temporal and geographical epidemiology of HBS in Belgium and the Netherlands, both at the cow and herd levels. From 2009 to 2022, based on necropsy records, an increasing trend of the yearly incidence of HBS in necropsied adult dairy cattle can be observed. While before 2018, yearly incidence of HBS was rarely more than 2%; during the most recent three years almost 1 in 30 necropsied mature dairy cows died because of HBS. At the herd level, estimated incidence rates increased even more in this time period; this was also the case when herd size was taken into account. On a monthly basis, HBS seems to occur more frequently in certain months. It was most prevalent in March, October, November and December. Fall appears to be the season with the highest incidence of HBS, being 40.1% higher than the average of the other seasons, and significantly higher than spring and summer. This differs from data published by the United States Department of Agriculture, indicating most cases occur in winter (38.1%) [17] Altogether, these data suggest that HBS is a relevant and possibly emerging problem in the dairy industry in Belgium and the Netherlands. 

There seems to be a correlation between higher herd size and occurrence of HBS. At the herd level, estimated incidence was twice as high in the Netherlands compared to Belgium. This could be secondary to several reasons, but the difference in herd size (Dutch herds are almost double the size of Belgian herds) is most likely an underlying factor. A higher herd size might be a cause for an increased estimated incidence, since larger herds have more animals at risk in each herd; also, other feeding and management practices or animal genetics might play a role. On the other hand, a higher herd size might be a source for bias, as necropsy submission behavior might be different between larger and smaller dairy farms. In addition, the 35 surveyed Flemish herds had a significantly higher herd size and rolling herd average compared to the Flemish average. Also in other studies, having a larger herd size was associated with HBS [12,17]. Larger dairies tend to be more professional and higher producing than smaller ones [18]. Management practices stimulating high milk production are also associated with HBS occurrence [12]. In fact, the evolution of the dairy industry in Belgium and the Netherlands, to larger and higher producing operations in the past decennia, might explain the increasing yearly incidence of HBS. Future research should investigate risk factors for HBS in larger dairy operations.

A second aim of this study was to describe characteristics of herds and cows with HBS. The vast majority of cows with HBS in this study were mature dairy cattle (97.9%, 277/283), which is typical for this disease [1]. However, six animals (2.1%) were beef cattle, with half being males. Almost all animals with known breeds were Holstein Friesians, although two animals were Belgian Blue. This is the first time HBS is documented in this breed. Holstein Friesian is the most common dairy cattle breed in Belgium. Brown Swiss or derived breeds are overrepresented in two studies [2,3]. Nevertheless, no animals of this breed are listed in this study, most likely because it is a very uncommon breed in Belgium, and only 42 Belgian cases of the breed were known. For 27 Flemish cows with HBS, more data was available. Most of these animals (84.6%) were second or later lactation animals, which is similar to other studies [11,12]. However, concerning the lactational stage, 61.1% (16/26) were in more than 100 days lactation, which is different to other reports where HBS was mainly reported in the first 100 days of lactation [11,12]. In 73.1% (19/25) of cases, animals died within 48 h, which confirms HBS has a very rapid clinical course [1]. In addition, observation of greedy eating behavior was reported in one study to be associated with HBS [5]. Only 15.4% (4/26) of animals were described by the producer as being greedy eaters, but this is very subjective.

On average, RHA and herd size of the 35 Flemish dairies with HBS were higher than the Flemish average at the time of occurrence. This is comparable to what is described by Berghaus et al. (2005), that HBS is more common in larger, higher-producing operations [12]. However, this is not an absolute prerequisite because 25.7% (9/35) and 37.1% (13/35) had an RHA and herd size, respectively, lower than the Flemish average. In the majority (74.3%, 26/35) of herds, it was the first confirmed case of HBS. This is compatible with the fact only 7.0% (19/272) of herds with HBS (according to necropsy data), had more than one case of HBS confirmed at a necropsy facility. However, 25.7% (9/35) of herds that participated in the survey had more suspected cases of HBS. It is most likely that once producers and their veterinarians think to recognize HBS clinically, no necropsy is done. In six out of the nine dairies with multiple cases, all cases occurred within a one-year time period. In multiple case operations, the time interval between consecutive cases was highly variable ranging from 1 day to 8 months, and averaged 137 days. A new corn silage or grass silage was opened in the past 3 weeks before HBS occurrence in 30.3% (10/33) and 33.3% (11/33) of operations, respectively. Taking into account that these dairies averaged 2.72 corn and 3.53 grass silages on each farm (15.7% and 20.3% of the year within 3 weeks before opening, respectively), this suggests that this could be a relevant finding and that the opening of a new grass or corn silage could be a risk factor. In the majority of dairy operations (69.7%), a feed change had also occurred in the past 3 weeks. This might also explain why HBS was more common in fall, a period where new corn silages are often opened and rations are adapted to the composition of the newly opened silage. Whether this apparent association has any causal basis requires further evaluation. However, if the opening of the silage was a major risk factor, multiple and temporal clustering of cases in a herd would be expected.

## 5. Conclusions

In conclusion, this study indicates that HBS is an important disease in adult dairy cattle in Belgium and the Netherlands, with an increasing incidence at both cow level, and even more at herd level, in the past decade. Both the increasing incidence over the years, as well as the higher incidence of HBS in the Netherlands compared to Belgium at herd level, could be related to the evolution of the dairy industry to larger and higher producing operations. Also, HBS was more frequent in fall, which could be associated with the opening of new silage and feed changes, which are common in that time of the year. In addition, based on the detailed observation of 35 herds, HBS seems to manifest itself as solitary cases, and if multiple cases occurred, the time interval between cases was highly variable. The majority of cows with HBS (61.1%; 16/26) were in more than 100 days lactation, which was different than previously described. Data in this study helps to better frame HBS as an impactful disease in dairy cattle.

## Figures and Tables

**Table 1:** Characteristics of Flemish herds with hemorrhagic bowel syndrome diagnosed between 2013–2022.

Variable	n/tot n	%		Mean ± SD
Rolling herd average (RHA)				10,199 ± 958.4
<9400 L	9/35	25.7		
9400–10,000 L	9/35	25.7		
>10,000 L	17/35	48.6		
Herd size (mature animals)				129.9 ± 32.7
<75	6/35	17.1		
75–150	19/35	54.3		
>150	8/35	22.3		
Occurrence of HBS on dairy				
Dairies with 1st HBS case ever	26/35	74.3		
Dairies with additional suspected HBS cases	9/35	25.7		
Dairies with multiple cases	9/35	25.7		
Dairies with multiple cases over >1 year	3/35	8.6		
	Mean	SD	n	Range
HBS cases in multiple case dairies	2.7	0.9	9	2–4
Interval between consecutive HBS cases (days)	137.6	114.3	10	1–240
Interval between first—last HBS case (days)	258.6	255.3	9	1–722
Suspected cases in each dairy with suspected HBS cases	0.8	2.5	9	1–9
HBS occurrences associated with recent (≤3 weeks) change in nutritional composition	n/tot n	%		
Feed change ^1^	23/33	69.7		
Opening of a new corn silage bunker	10/33	30.3		
Opening of a new grass silage bunker	11/33	33.3		
Other feed changes ^2^	13/33	39.4		

^1^ Feed change is defined as opening of a new silage bunker and/or other feed changes. ^2^ Other feed changes is defined as replacement, addition or any change in the amount of feed components, but not opening of a new silage bunker.

**Table 2:** Characteristics of Flemish cows with hemorrhagic bowel syndrome diagnosed between 2020–2022.

Variable	Mean	SD	Range
Age (year)	3.9	3.9	2–8
Lactation number	2.6	2.6	1–6
Days in milk	163.8	114.7	28–366
	n/total n	%	
First lactation cows	4/27	14.8	
>1 laction cows	23/27	85.2	
Fresh cows (0–21 d)	0/26	0.0	
Peak lactation (21–100 d)	10/26	38.5	
Mid lactation (101–200 d)	6/26	23.1	
Late lactation (>200 d)	10/26	38.5	
Interval observation disease–natural death			
<24 h	7/25	28.0	
24–48 h	12/25	48.0	
>48 h	6/25	24.0	
Spontaneous death	13/25	52.0	
Observation of bloody feces	20/26	76.9	

## Data Availability

Restrictions apply to the availability of these data. Data was obtained from Royal GD and are available from the authors with the permission of Royal GD.
